# Neonatal preintubation sedation: a national survey in Malaysia

**DOI:** 10.1186/s13104-015-1653-3

**Published:** 2015-11-09

**Authors:** Amar–Singh HSS, Sharon Linus-Lojikip, Zarena Ismail, Nurul-Huda Ishahar, Siti-Suhaila Yusof

**Affiliations:** Pediatric Department and Clinical Research Centre Perak, Hospital Raja Permaisuri Bainun, Jalan Hospital, 30990 Ipoh, Perak Malaysia; Clinical Research Centre Perak, Hospital Raja Permaisuri Bainun, Jalan Hospital, 30990 Ipoh, Perak Malaysia; College of Nursing, Ipoh, Perak Malaysia

**Keywords:** Preintubation sedation, Neonates, NICU, Malaysia, Policy

## Abstract

**Background:**

There is a shift of practice towards administering sedation in neonates around the world. At the present moment, there is no available data or literature on the practice of sedation before intubation of neonates in Malaysia thus, evaluation of these practice was not possible. This study was conducted to evaluate neonatal preintubation sedation practice and the availability of neonatal preintubation sedation policy in government, university and private Malaysian Neonatal Intensive Care Units (NICUs) in 2007.

**Methods:**

All 43 NICUs in Malaysia were identified and approached to participate in the study. Phone interviews with doctors’ in-charge of NICUs were conducted in 29 governments, 3 universities and in 7 private NICUs.

**Results:**

Only 7 NICUs had written policy on neonatal preintubation sedation use. Seventy-seven percent and 97.4 % of NICUs used sedation during emergency intubation and during planned intubation respectively. Sixty seven percent used either morphine or midazolam with no preference of either drug.

**Conclusion:**

This study showed a significant proportion of NICUs used sedation during emergency or planned intubation. However, the majority does not write policy on neonatal preintubation sedation use (82.1 %). The types and drug administration methods are not standardized in all of the NICUs. This will require a standard national written policy to be developed.

## Background

Research findings have demonstrated that neonates are capable of feeling pain [1–3]. As a result, there is a shift of practice, among pediatric professionals around the world, towards administration of procedural analgesics and sedation in neonates. Intubation is one of the many painful and distressing procedures that most neonates have to undergo when admitted into Neonatal Intensive Care Units (NICUs) [2–6]. Pain and physiological distress associated with invasive procedures [7, 8] may potentially increase the morbidity in these neonates. Pre-medicated intubation in neonates is deeming more humane, safer and more effective method than the previously thought conventional method; awake intubation [1, 4, 9].

There has been a substantial growth in the number of neonatal units in the United Kingdom (UK) that provide premedication for non-emergent newborn intubation since 1998 [10, 11]. The availability of written policy and guidelines concerning premedication prior to neonatal intubation in the UK has also increased from 14 % in 1998 to 77 % in 2009 [10, 11]. Now, there is no available data or literature on the practice of sedation before intubation of neonates in Malaysia. This study was to look at the practice and policy availability for neonatal preintubation sedation in the Malaysian NICUs.

## Methods

This cross-sectional study was conducted via phone interview from October to November in 2007. All established NICUs from government, university and private hospitals in Malaysia, were identified and included in the study. There were no exclusion criteria. All the specialist in-charge or the pediatric trainee in-charge of all the NICUs were approached via phone and were invited to participate in the study. Those who agreed, gave their verbal consent and were then interviewed using a structured questionnaire on the same day. The names of interviewed staff and hospitals remained confidential at all times.

The structured questionnaire was designed based on a literature search on the use of preintubation sedation in neonates. The interview included request on the following information about the department’s routine practice from the interviewee: availability of written policy on preintubation sedation use in neonates in the department, the standard use of sedative agents in emergency or planned intubation, reasons for preintubation sedation use, types of sedative agents and other drugs used, methods of administering preintubation sedation, category of staff allowed to give the sedation and selection criteria for sedation use in neonates.

Fisher exact test was used in the analysis. A p value of less than 0.05 was considered statistically significant. Clinical Research Centre Perak funded this study and ethical approval was obtained from the Medical and Research Ethics Committee, Ministry of Health Malaysia.

## Results

### Characteristic of study participants

All 43 NICUs in Malaysia were approached and 39 (90.7 %) NICUs agreed to participate in the study. One NICU from the government and three from the private sector declined participation due to administrative obstacles and busy clinical duties. Of the 39 participating NICUs, 29 (74.0 %) were from government, three (8.0 %) were from university and seven (18.0 %) were from private hospitals. One neonatologist, 25 pediatricians, and 13 pediatric trainees participated in the study and were interviewed. Although the researchers targeted mainly pediatricians or neonatologists, they were not always available and pediatric trainees were interviewed as a proxy.

### Policy and practice of neonatal preintubation sedation

Only seven (17.9 %) NICUs had a written policy on preintubation sedation for neonates (six government NICUs and one university NICU). Thirty-eight (97.4 %) NICUs used sedation for planned intubation and one private NICU practiced awake planned intubation. Thirty (76.9 %) of the NICUs also used sedation for emergency intubation which included all university NICUs, 25 government NICUs and two private NICUs. Government and university NICUs were significantly more likely to use sedation during emergency intubation than private NICUs (Fisher exact test = 0.011). Despite routine use of sedation, 20 (51.3 %) NICUs would evaluate the condition of the neonate first before deciding on its use. Criteria for decision making included neonatal signs of distress, struggling or fighting, presence of sepsis and prematurity.

### Reasons for sedation practice

Majority of the respondents stated the reasons for giving sedation during neonatal planned intubation was “to facilitate the process of neonatal intubation” (27, 50.0 %). This is followed by “required for pain relief” (25, 45.0 %) and others (2, 4.0 %). The reason given for practicing awake planned intubation by a private NICU was unaware of the need for neonatal preintubation sedation. “Limited time” (10, 83.3 %) and “condition of neonate is not stable” (2, 16.3 %) were the reasons given for not using preintubation sedation during neonatal emergency intubation.

### Agent types, administration routes and personnel for preintubation sedation

The use of sedative agents varied across all NICUs and they were used either as single agent or in combination. The commonest sedative agents used were either morphine or midazolam (Table 1). Nine (31.0 %) NICUs used muscle relaxants during intubation (six government and three private NICUs). The most commonly used method for administering preintubation sedation is bolus intravenous route; thirty-six (92.0 %) NICUs used this method. Three (8.0 %) other NICUs used other methods (intravenous infusion, intranasal and buccal routes). The personnel allowed to use sedation during neonatal intubation were mainly specialists or pediatric trainees in all three types of hospitals. The other personnel occasionally allowed were house officers and trained neonatal nurses (Table 2).

**Table 1:** Types of sedative agents used by different NICUs

Types of sedative agents used	Number of responses “Yes” to types of sedative agents used by different NICUs*			Total number of responses
	Government NICU	University NICU	Private NICU	
Midazolam alone	4	1	3	8
Morphine alone	3	0	1	4
Either morphine or midazolam (no preference)	21	2	3	26
Combination (morphine and midazolam combined when giving the sedation)	1	0	0	1
Other drugs (chloral hydrate, fentanyl, ketamine)	3	0	0	3

*More than one response is possible

**Table 2:** Level of personnel allowed using sedation during neonatal intubation by different NICUs

Level of personnel	Number of responses “Yes” to level of personnel allowed to use sedation during neonatal intubation by different NICUs*			Total number of responses
	Government NICU	University NICU	Private NICU	
Specialist	29	0	5	34
Medical officer	29	3	1	33
House officer	1	0	0	1
Trained neonatal nurse	0	0	1	2

*More than one response is possible

## Discussion

Neonatal preintubation sedation was widely practiced in the Malaysian NICUs at the time of the study. Although widely practiced, these may not proportionally reflect the awareness or knowledge on the need for neonatal intubation pain relief among the pediatric professionals in general. This was demonstrated by the large percentage of the NICUs that reported the use of preintubation sedation to ‘facilitate the process of intubation’ (50.0 %), rather than to alleviate pain. In addition, one NICU practiced awake or conscious planned intubation for lacking the awareness on the need for preintubation sedation in neonates.

The best choice of preintubation medication agents or combination of agents is still unclear [12]. Current evidence supports the use of either an analgesic or hypnotic medication and that sedatives alone should be avoided. The wide range of practices in Malaysian NICUs is of concern as many used sedatives alone and others drugs of questionable value (chloral hydrate and ketamine). Some used either analgesic or sedatives without preference. This supports the need for guidance and a neonatal preintubation sedation policy.

We compared the premedication practice and policy availability for neonatal intubation from various countries based on available published studies that used similar study methodology (Table 3). We found that the overall percentage for neonatal premedication intubation practice was higher in the Malaysian NICUs as compared to NICUs from other countries before the year 2007 (Table 3). Availability of neonatal premedication intubation policy in NICUs in Malaysia however was lower (17.9 %) compared to France (60 % in the same year, 2007) and United Kingdom (70 % in a year later, 2008).

**Table 3:** Practice of premedication use and policy coverage for neonatal intubation in various countries based on available published studies

No	Author	Chronology publication Year	Country	Number of NICU sampled	NICU level of care	Percentage of premedication use by NICU (%)	Percentage of units with policies & protocols (%)
1	Ziegler et al. [9]	1992	USA	101	III	3.0 %	Not reported
2	Whyte et al. [10]	2000	United Kingdom	239	II & III	37.0 %	14.0 %
3	Simon et al. [13]	2004	France	75	III	37.0 %	Not reported
4	Lago et al. [14]	2005	Italy	90	II & III	13.0 %	25.0 %
5	Walter-Nicolet E et al. [17]	2007	France	58	Not reported	Not reported^#^	60.0 %
6	This study	2007	Malaysia	39	II & III	97.4 %	17.9 %
7	Mckechnie et al. [15]	2008	United Kingdom	192	II & III	Not reported	70.0 %
8	Kelleher et al. [16]	2009	United Kingdom	221	II & III	93.0 %	76.0 %
9	Chaudhary et al. [11]	2009	United Kingdom	50	III	90.0 %	77.0 %
10	Wheeler et al. [18]	2012	Australia and New Zealand	28 NICUs and two neonatal emergency transport services	All the tertiary NICUs and neonatal emergency transport services	100.0 %	80.0 %
11	Y Singh et al. [19]	2012	United Kingdom	44	Tertiary neonatal units	100.0 %	91.0 %

^#^This study reported that 74.0 % of the newborns received a sedative and/or an analgesic before being intubated

This study evaluated the policy availability and practice for neonatal preintubation sedation in all NICUs in Malaysia for the first time in 2007. The findings however may not reflect the current practices for neonatal preintubation sedation in the Malaysian NICUs as this study was completed in 2007. Telephone survey methodology and using trainees as proxies to elicit information for the NICU practices in some cases, also impaired the information validation for accuracy and actual practice. Despite the limitations, our study provides an overview of neonatal preintubation sedation practice in Malaysia. In the future, re-evaluation of current practice and policy availability for neonatal preintubation sedation should utilizes study methodology that can verify actual practices.

## Conclusion

This study has shown that a significant proportion of the government, university and private NICUs in Malaysia use sedation during planned or emergency neonatal intubation. However, the majority did not have a formal written policy for neonatal sedation use. Half of the NICUs reported to use sedation to facilitate neonatal intubation rather than for pain relief. The sedative agent types and administration routes varied widely across all types of NICUs. A standardization of practices with a national policy adopted by all types of NICUs would be desirable.

## Abbreviations

NICU: neonatal intensive care unit
NICUs: neonatal intensive care units
